# Multiple secretoglobin 1A1 genes are differentially expressed in horses

**DOI:** 10.1186/1471-2164-13-712

**Published:** 2012-12-19

**Authors:** Olivier Côté, Brandon N Lillie, Michael Anthony Hayes, Mary Ellen Clark, Laura van den Bosch, Paula Katavolos, Laurent Viel, Dorothee Bienzle

**Affiliations:** 1 Departments of Pathobiology, University of Guelph, Stone Road, Guelph, ON, Canada; 2 Departments of Clinical Studies, University of Guelph, Stone Road, Guelph, ON, Canada; 3 Present address: Genentech, South San Francisco, CA, 94080, USA

**Keywords:** CC10, Clara cell, Clara cell secretory protein, End-point limiting dilution PCR, Horse, Immunohistochemistry, Long-range PCR, Recurrent airway obstruction, Uteroglobin

## Abstract

**Background:**

Secretoglobin 1A1 (SCGB 1A1), also called Clara cell secretory protein, is the most abundantly secreted protein of the airway. The *SCGB1A1* gene has been characterized in mammals as a single copy in the genome. However, analysis of the equine genome suggested that horses might have multiple *SCGB1A1* gene copies. Non-ciliated lung epithelial cells produce SCGB 1A1 during inhalation of noxious substances to counter airway inflammation. Airway fluid and lung tissue of horses with recurrent airway obstruction (RAO), a chronic inflammatory lung disease affecting mature horses similar to environmentally induced asthma of humans, have reduced total SCGB 1A1 concentration. Herein, we investigated whether horses have distinct expressed *SCGB1A1* genes; whether the transcripts are differentially expressed in tissues and in inflammatory lung disease; and whether there is cell specific protein expression in tissues.

**Results:**

We identified three *SCGB1A1* gene copies on equine chromosome 12, contained within a 512-kilobase region. Bioinformatic analysis showed that *SCGB1A1* genes differ from each other by 8 to 10 nucleotides, and that they code for different proteins. Transcripts were detected for *SCGB1A1* and *SCGB1A1A*, but not for *SCGB1A1P*. The *SCGB1A1P* gene had most inter-individual variability and contained a non-sense mutation in many animals, suggesting that *SCGB1A1P* has evolved into a pseudogene. Analysis of *SCGB1A1* and *SCGB1A1A* sequences by endpoint-limiting dilution PCR identified a consistent difference affecting 3 bp within exon 2, which served as a gene-specific “signature”. Assessment of gene- and organ-specific expression by semiquantitative RT-PCR of 33 tissues showed strong expression of *SCGB1A1* and *SCGB1A1A* in lung, uterus, Fallopian tube and mammary gland, which correlated with detection of SCGB 1A1 protein by immunohistochemistry. Significantly altered expression of the ratio of *SCGB1A1A* to *SCGB1A1* was detected in RAO-affected animals compared to controls, suggesting different roles for SCGB 1A1 and SCGB 1A1A in this inflammatory condition.

**Conclusions:**

This is the first report of three *SCGB1A1* genes in a mammal. The two expressed genes code for proteins predicted to differ in function. Alterations in the gene expression ratio in RAO suggest cell and tissue specific regulation and functions. These findings may be important for understanding of lung and reproductive conditions.

## Background

The mammalian airway epithelium is composed of heterogeneous cell populations grouped into three main types according to morphology and function: basal, ciliated, and secretory cells [1]. Further, eight morphologically defined subtypes are recognized, including non-ciliated, cuboidal and secretory Clara cells [2]. Clara cells synthesize the most abundantly secreted protein in the airway surface fluid, secretoglobin family 1A, member 1 (SCGB 1A1) [3]. The list of names attributed to SCGB 1A1 is extensive and includes Clara Cell Secretory Protein (CCSP), uteroglobin, blastokinin, Clara cell 10 kDa protein (CC10), CC16, polychlorinated biphenyl-binding protein (PCB-BP), and urine protein-1 (UP1). SCGB 1A1 has been ascribed many functions including binding of lipophilic substances, inhibition of leukocyte recruitment, inhibition of phospholipase A_2_, and other anti-inflammatory roles (reviewed in [4,5]).

SCGB 1A1 is consistently expressed at high levels in the lung of most mammalian species. More specifically, studies in the horse showed that SCGB 1A1 is detected in non-ciliated lung epithelial cells, but not in goblet or ciliated epithelial cells [6]. SCGB 1A1 comprises 2 to 12% of bronchoalveolar lavage (BAL) fluid proteins [7,8], and is considered to be an important component of proteins protecting the pulmonary epithelium against deleterious inhaled environmental substances [9]. It was previously reported that horses with recurrent airway obstruction (RAO), an asthma-like chronic inflammatory condition affecting mature individuals, have ultrastructural changes in Clara cells [6], decreased lung *SCGB1A1* gene expression [6,10] and reduced BAL fluid SCGB 1A1 concentration [6]. Expression of *SCGB1A1* has also variously been described in extra-pulmonary tissues from diverse species. In horses, *SCGB1A1* transcripts were present in uterine and prostatic tissues and absent in liver, kidney, heart, spleen, thyroid, pituitary and adrenal gland tissues [11].

A single *SCGB1A1* gene has been described in the genome of multiple mammals, including rabbit, rat, mouse, monkey, and human [12-15]. The general structure of the *SCGB1A1* gene includes two introns and three exons coding for a small secreted protein of ~70 amino acids. This organizational structure is remarkably conserved between species; however, the length of the *SCGB1A1* genomic locus fluctuates [12-17]. In horses, the first reported sequence was described as a unique cDNA and was ascribed to a single gene [11]. However, the recent availability of the complete *Equus caballus* genome sequence provided evidence of three highly similar *SCGB1A1* gene sequences on chromosome 12, suggesting the horse has diverged from the “single copy” *SCGB1A1* consensus. Two distinct SCGB 1A1 protein products were also identified in uterine fluids during early pregnancy [18], further implying that more than one *SCGB1A1* gene may be transcribed and translated.

Considering that horses appear to have multiple similar, but not identical, *SCGB1A1* gene copies, and that total SCGB 1A1 levels are decreased in the lung of horses with RAO, we hypothesized that *SCGB1A1* variants may be differentially expressed and have different functions. Herein, we report on three distinct copies of the *SCGB1A1* gene in horses. We developed assays to distinguish each gene, determined tissue- and copy-specific gene expression, and evaluated cell-specific presence of the SCGB 1A1 protein. We further determined that horses with RAO have an abnormal expression ratio of different *SCGB1A1* genes.

## Results

### Identification and localization of *SCGB1A1* genes

**Figure 1:**
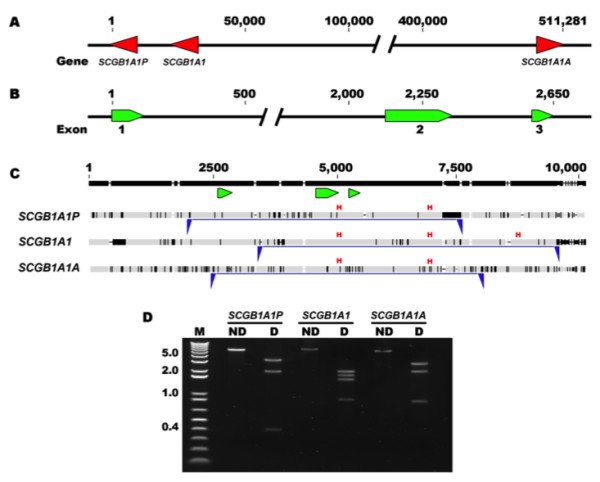
**Schematic representation of *SCGB1A1 *sequencing strategy and enzymatic restriction analysis.** (**A**) The partial genomic sequence of *Equus caballus* chromosome 12 (based on EquCab2.0, NW_001867370.1) encompassing the three predicted equine *SCGB1A1* genes (red triangles). *SCGB1A1P* and *SCGB1A1* are in reverse orientation, while *SCGB1A1A* is in forward orientation. The chromosome 12 region (original bases 2,788,223 to 3,299,503) includes 511,281 bp bordered by the *SCGB1A1P* stop codon guanine (position 1) and the *SCGB1A1A* stop codon guanine (position 511,281). *SCGB1A1P* is most proximal to the centromere. (**B**) Predicted structure of an individual *SCGB1A1* gene, each containing approximately 2,650 bp, including 3 small exons (green arrows) and 2 introns. (**C**) Multiple sequence alignment of the three predicted *SCGB1A1* genes. The LR-PCR amplification strategy is outlined in blue. Each primer (blue triangle) is specific for a single copy enabling amplification of three distinct PCR products (blue line). Exons are displayed in green under the consensus sequence (black). (**D**) Restriction enzyme digestion analysis. Non-digested amplicons for *SCGB1A1P*, *SCGB1A1* and *SCGB1A1A* were 5,559, 6,029 and 5,442 bp in size, respectively. Upon *HindIII* digestion, *SCGB1A1P* was detected by 3166, 1942 and 464 bp, *SCGB1A1* by 1942, 1,717, 1563, and 891 bp, and *SCGB1A1A* by 2675, 1942, and 899 bp fragments. H (red), *HindIII* restriction site; M, 1 Kb + DNA ladder; ND, Non-digested; D, Digested.

Basic Local Alignment Search Tool (BLAST) was used to determine sequence similarity between the most recent high-quality equine chromosome-12 genomic sequence (EquCab2.0; NW_001867370.1) [19] and the previously described equine *SCGB1A1* precursor mRNA (AY885564.1). Nine BLAST hits were identified at positions 2788223-2788256 (100% identity, e-value 2^-10^), 2788555-2788748 (99% identity, e-value 2^-95^), 2790815- 2790869 (96% identity, e-value 9^-19^), 2810573- 2810606 (100% identity, e-value 2^-10^), 2810905-2811098 (99% identity, e-value 4^-97^), 2813166- 2813220 (98% identity, e-value 2^-20^), 3296852- 3296906 (98% identity, e-value 2^-20^), 3298978-3299171 (97% identity, e-value 8^-89^), and 3299470-3299503 (94% identity, e-value 4^-07^), consistent with the presence of three *SCGB1A1* gene copies on chromosome 12, each encompassing three exons (hits). The three predicted copies were contained within a 512-kilobase (kb) region, with two copies positioned in reverse orientation and one in forward orientation (Figure 1A).

A partial sequence including a large part of the adjoining 5’ and 3’ non-coding DNA was extracted from the EquCab2.0 sequence for each predicted copy (~10 kb/sequence) and analyzed by multiple sequence alignment. Bioinformatic analysis confirmed that each gene had comparable exon/intron organization, and covered about 2,650 base pairs (bp) of genomic DNA (Figure 1B). A high degree of pairwise identity (92.7%) was observed in large segments overlapping the *SCGB1A1* coding regions and 8,941 identical sites (87.8%) were found among the three genes, suggesting that *SCGB1A1* genes developed from an intrachromosomal triplication event. The pairwise identity increased to 97.8% and the number of identical sites rose to 96.7% upon alignment of the predicted complementary DNA (cDNA) sequences. However, *SCGB1A1* genes differed from each other by 8 to 10 nucleotides, and were expected to produce different proteins. Therefore, the distinct genes were termed *SCGB1A1P*, *SCGB1A1*, and *SCGB1A1A*; with *SCGB1A1P* located most proximal to the centromere. These novel sequences were deposited in GenBank with the following accession numbers: JQ951929, JQ951930 and JQ951931.

### Isolation and characterization of *SCGB1A1* genomic sequences

A long-range (LR)-PCR strategy was developed to amplify individual full-length *SCGB1A1P*, *SCGB1A1*, and *SCGB1A1A* genomic sequences, using three distinct gene-specific primer sets (Figure 1C). The size of the different LR-PCR products ranged from 5.4 to 6.0 kb. Samples from a total of 24 animals were used for amplification, purification, and identification of the *SCGB1A1P* sequence, compared to *SCGB1A1* and *SCGB1A1A* sequences, which were accurately documented by the examination of 12 sequences due to a reduced amount of polymorphisms between individuals analyzed. The identity of all PCR fragments was further evaluated by restriction enzyme digestion assay (Figure 1D), which confirmed presence of three different *SCGB1A1* genes in each animal assessed.

**Figure 2:**
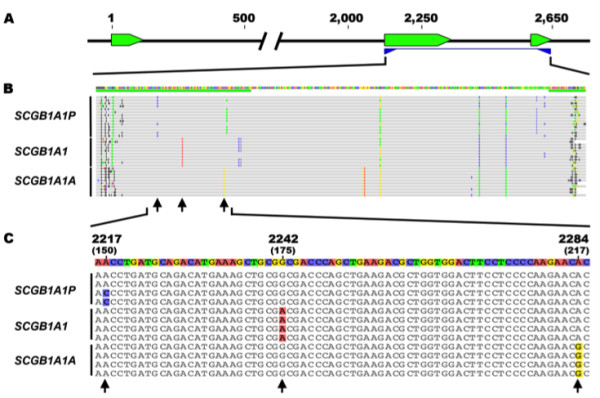
**Characterization of *SCGB1A1 *genomic sequences.** (**A**) A partial *SCGB1A1* genomic sequence was amplified by nested PCR using purified LR-PCR products as template. Primers (blue triangles) were designed to target *SCGB1A1* exon 2 to 3 (green boxes), coding for the complete mature secreted protein. (**B**) Multiple sequence alignment was performed using the nested PCR products from *SCGB1A1P*, *SCGB1A1* and *SCGB1A1A*. Samples from 24 animals were analyzed to establish the *SCGB1A1P* consensus sequence, and 12 samples for *SCGB1A1* and *SCGB1A1A*. (**C**) A region of the SCGB1A1 exon 2 gene sequence showing the three individual single nucleotides (arrows) at positions 2217/2242/2284 (cDNA positions 150/175/217) used as internal markers for subsequent gene-specific identification (*SCGB1A1P* = C/A--G--A; *SCGB1A1* = A--A--A; *SCGB1A1A* = A--G--G).

The genomic region coding for the complete mature secreted protein (including exons 2 and 3) was subsequently targeted by nested PCR, using the previously purified LR-PCR products as template (Figure 2A). Hence, 24, 12 and 12 amplicons were generated in duplicate from *SCGB1A1P*, *SCGB1A1*, and *SCGB1A1A* full-length sequences, respectively. Each PCR product was individually analyzed by electrophoresis, purified and sequenced, using both forward and reverse sequencing. The resultant 96 *SCGB1A1P*, 48 *SCGB1A1* and 48 *SCGB1A1A* sequences were subjected to multiple sequence alignment. A high level of agreement was found between the different sequences from each gene, with pairwise identity consistently greater than 98%. A consensus sequence was determined for *SCGB1A1P*, *SCGB1A1*, and *SCGB1A1A* (Figure 2B). Bioinformatic analysis revealed a combination of three non-contiguous single nucleotide differences that conferred a gene-specific “signature” sequence at positions 150, 175 and 217 on the cDNA map (Figure 2C). As shown in Figure 2C, different sites comprising the signature were C/A--G--A, A--A--A, and A--G--G in *SCGB1A1P*, *SCGB1A1*, and *SCGB1A1A*, respectively. These differences were subsequently used for individual *SCGB1A1* transcript identification.

### Isolation and characterization of *SCGB1A1* transcripts

**Figure 3:**
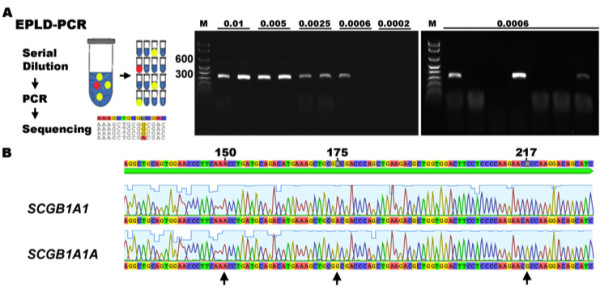
**End-point limiting dilution PCR strategy.** (**A**) Left: Schematic illustration of EPLD-PCR. Center: Serial cDNA dilutions (ng/μl) were tested to determine an approximate limiting dilution concentration of template. Right: Each selected limiting dilution was further tested to confirm the PCR efficiency. Here, 3 out of 7 PCR amplifications were positive for the specific 256-bp amplicon, confirming a <50% expected value (efficiency = 33%). (**B**) EPLD-PCR amplicon sequences. Only *SCGB1A1* and *SCGB1A1A* were identified on the chromatograms using the gene-specific signature (arrows). No *SCGB1A1P* cDNA was identified. M, 100-bp marker; concentrations are indicated in ng/μL.

To evaluate the transcriptional state of activation of each *SCGB1A1* gene, end-point limiting dilution (EPLD)-PCR was performed with serially diluted cDNA preparations from adult equine lung (n = 3) and uterus (n = 3) tissues. Tissues were selected on the basis of their strong total *SCGB1A1* transcript expression [11]. Primers were developed in conserved regions of the genes to avoid gene-specific preference during the amplification process. Each PCR assay was performed using a defined amount of an optimized limiting cDNA concentration as template (Figure 3A). The dilution that resulted in detectable amplification in less than 50% of the reactions was considered to be limiting. At this concentration, the DNA target was assumed to reflect a Poisson distribution, suggesting that 50% of the reactions did not contain cDNA template and therefore detectable products originated from a single template.

A total of 212 PCR products were amplified from 665 reactions (32% efficiency) using lung cDNA limiting dilutions as template (42-124 amplicons/animal). An additional 86 products were generated from 175 reactions (49% efficiency) using uterine cDNA (28-29 amplicons/animal). Each individual PCR product was purified, sequenced (forward), and identified by the *SCGB1A1* signature sequences (Figure 3B). In lung samples, 30% of products were identified as *SCGB1A1* cDNA (A--A--A) and 70% as *SCGB1A1A* (A--G--G). Likewise, in uterus samples, 56% were identified as *SCGB1A1* and 44% as *SCGB1A1A*.

From the 298 amplicons identified, 20 *SCGB1A1* and *SCGB1A1A* cDNAs were randomly selected for further characterization. Each sample was re-submitted for sequencing in both forward and reverse directions, and results were aligned to determine the cDNA sequence delimited by the start and stop codons. Subsequently, *SCGB1A1* and *SCGBA1A* cDNA consensus sequences were obtained by alignment of the 20 gene-specific sequences (accession numbers JQ906259, JQ906260, JQ906261, Additional file 1: Figure S1). As expected, strict nucleotide identity was observed between an individual cDNA and the corresponding genomic sequence. However, several disagreements were observed upon comparison with the predicted EquCab2.0 cDNA sequences. Among samples in this study, the partial *SCGB1A1* cDNA consensus sequences displayed a non-conservative substitution at position 19 (A to G) and two variable codons (Variant A, TTA or variant B, CTC) at positions 232 to 234. The latter variants both code for the production of a leucine residue. Similarly, our *SCGB1A1A* consensus sequence showed 5 non-conservative substitutions at position 65 (A to G), 78 (C to A), 81 (T to G), 175 (A to G), and 217 (A to G) compared to the EquCab2.0 *SCGB1A1A* predicted cDNA sequence. Single-nucleotide insertions or deletions were not detected.

Surprisingly, *SCGB1A1P* cDNA was not detected by EPLD-PCR. Since complete genomic/cDNA nucleotide identity was observed for *SCGB1A1* and *SCGB1A1A*, *SCGB1A1P* similarly was assessed using the predicted cDNA sequences extracted from EquCab2.0 and our consensus genomic sequences (accession numbers JQ951929, JQ951930, JQ951931, Additional file 1: Figure S1). Analysis revealed 98% pairwise identity between the predicted cDNA and the genomic DNA (272/276 nucleotides). Three non-conservative substitutions were identified at positions 14 (T to G), 19 (G to A), and 220 (A to T). Most strikingly, the A to T substitution detected at position 220 in the variant sequences B and C represented 54% of the horses analyzed (13/24), independent of their genetic background. This polymorphism is expected to replace an AAG codon encoding lysine, to a TAG codon, encoding a stop codon. Therefore, these results demonstrate the presence of a *SCGB1A1P* gene variant that may encode a truncated protein in a large proportion of animals.

### *SCGB1A1* copy- and tissue-specific expression pattern

**Figure 4:**
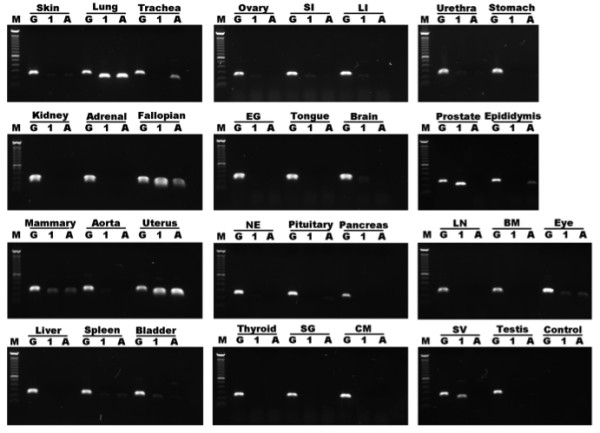
**Representative sample of sqRT-PCR evaluation of *SCGB1A1 *transcript levels in various equine tissues. ***SCGB1A1* (label “1”) and *SCGB1A1A* (label “A”) cDNAs were detected as 200 bp amplification bands. Equine *GAPDH* (label “G”) was amplified as an internal control (254 bp). Densely stained amplicons of *SCGB1A1* and *SCGB1A1A* cDNA were detected in lung, uterus, Fallopian tube and mammary gland tissues. Faint bands were present in multiple tissues including brain, pituitary, eye, nose epithelium, tongue, salivary gland, trachea, aorta, cardiac muscle, liver, spleen, small and large intestine, adrenal gland, kidney, skin, bladder, urethra, prostate, epididymis, seminal vesicle, testis and ovary. No PCR products were detected with cDNA from the eyelid gland, thyroid, bone marrow, pancreas, stomach and lymph node. SI, small intestine; LI, large intestine; EG, eyelid gland; NE, Nose epithelium; SG, salivary gland; CM, cardiac muscle; LN, lymph node; BM, bone marrow; SV, seminal vesicle.

Expression of *SCGB1A1* and *SCGB1A1A* specific mRNA was investigated by semi-quantitative reverse transcriptase (sqRT)-PCR in tissues from seven adult horses (two geldings, three mares and two stallions, Figure 4). This technique was selected to survey the expression pattern rather than determine precise expression levels, since a high degree of *SCGB1A1* variation was expected between different tissues. The results were categorized into three groups based on the amount of specific PCR product detected by density scanning after gel electrophoresis. A relative optical density (OD) value was attributed to each band, using glyceraldehyde dehydrogenase (*GAPDH*) amplicons as the reference value (Table 1).

**Table 1:** **Semi-quantitative analysis of*SCGB1A1*RT-PCR amplicons**

		**Relative amplicon density**			
**Expression category**	**Tissue**	***GAPDH***	***SCGB 1A1***	***SCGB 1A1A***	**n**
High	Lung	1.00	3.50 ± 0.94	3.65 ± 0.72	5
	Uterus	1.00	2.25 ± 0.59	2.02 ± 0.61	4
	Fallopian tube	1.00	2.67 ± 1.60	1.49 ± 1.59	3
	Mammary gland	1.00	0.67 ± 0.67	nd - 0.33	3
	Ovary	1.00	nd - 0.65	nd - 0.07	3
	Prostate	1.00	nd - 1.67	nd	2
Low	Aorta	1.00	0.42 ± 0.55	nd - 0.32	4
	Urethra	1.00	0.25 ± 0.38	0.01 ± 0.01	4
	Epididymis	1.00	nd - 0.07	0.16 ± 0.20	2
	Skin	1.00	nd - 0.49	0.04 ± 0.01	4
	Trachea	1.00	nd - 0.19	0.81 ± 0.73	4
	Eye	1.00	nd - 0.26	nd - 0.43	4
	Brain	1.00	0.09 ± 0.08	0.08 ± 0.02	4
	Nose epithelium	1.00	nd - 0.17	0.04 ± 0.03	4
	Bladder	1.00	0.06 ± 0.04	nd - 0.03	4
	Spleen	1.00	nd - 0.07	nd - 0.04	4
	Kidney	1.00	nd - 0.02	nd - 0.01	4
	Liver	1.00	nd - 0.02	nd - 0.01	4
	Tongue	1.00	nd - 0.05	nd	3
	Small intestine	1.00	0.02 ± 0.01	nd	4
	Large intestine	1.00	0.03 ± 0.02	nd	4
	Pituitary	1.00	nd - 0.01	nd - 0.02	4
	Salivary gland	1.00	nd - 0.01	nd	4
	Adrenal gland	1.00	nd - 0.01	nd	4
	Seminal vesicle	1.00	nd - 0.42	nd	2
	Testis	1.00	nd	0.02 ± 0.01	2
Absent	Eyelid gland	1.00	nd	nd	4
	Cardiac muscle	1.00	nd	nd	4
	Thyroid	1.00	nd	nd	4
	Stomach	1.00	nd	nd	4
	Pancreas	1.00	nd	nd	4
	Lymph node	1.00	nd	nd	4
	Bone marrow	1.00	nd	nd	3

Quantification of gene-specific *SCGB1A1* cDNA amplicon intensity relative to *GAPDH* using Image Lab™ software 2.0.1; nd, non-detected.

Results in the first group corresponded to cDNA samples that generated abundant PCR amplicons with high OD (>0.50) for both *SCGB1A1* genes. Samples from lung, uterus (non-pregnant), Fallopian tube, and mammary gland (non-lactating) consistently and reproducibly met this criterion.

The second group included cDNAs that produced a faint PCR product for either *SCGB1A1* or *SCGB1A1A*, and had relatively low (0.01 to 0.50) OD values. Brain, pituitary gland, eye, nose epithelium, tongue, parotid salivary gland, trachea, aorta, liver, spleen, small and large intestine (cecum), adrenal gland, kidney, skin, bladder, urethra, prostate, epididymis, seminal vesicle, testis and ovary were included in this group.

A third group included cDNAs that did not produce gel-detectable PCR amplicons for any sample tested, and therefore, were unsuitable for quantitative assessment. Eyelid gland, thyroid, bone marrow, cardiac muscle, pancreas, stomach and lymph node were considered negative for *SCGB1A1* expression.

### Quantification of *SCGB1A1* gene-specific expression level

**Figure 5:**
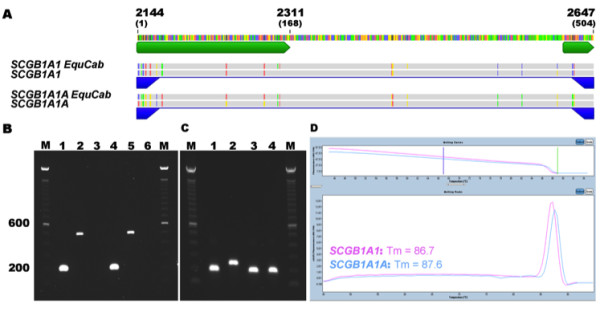
***SCGB1A1 *and *SCGB1A1A *qRT-PCR strategy.** (**A**) Multiple sequence alignment of the partial predicted *SCGB1A1* and *SCGB1A1A* EquCab2.0 genomic sequences as well as the *SCGB1A1* and *SCGB1A1A* sequences derived in this study. Two gene-specific primer sets (blue arrows) were designed at sites with the greatest amount of sequence variation (colored annotations above primers). Forward primers are positioned in exon 2 (long green arrow) and reverse primers in exon 3 (small green arrow). This region covers position 2,144 to 2,647 of *SCGB1A1* genes corresponding to a 504 bp genomic DNA section (brackets). (**B**) As expected, *SCGB1A1* was detected as a 200 bp PCR product using lung cDNA as a template (Lane 1), a 504 bp band using genomic DNA as template (Lane 2), and undetected with template omission (negative control, Lane 3). Similar findings were noted for *SCGB1A1A* (Lane 4, 5, and 6, respectively). (**C**) qRT-PCR products were analyzed after electrophoresis to assure cDNA quality and confirm the absence of genomic DNA contamination. Bands were expected at 203 bp for equine 18S (Lane 1), 254 bp for *GAPDH* (Lane 2), 200 bp for *SCGB1A1* (Lane 3) and *SCGB1A1A* (Lane 4). (**D**) Melting curve analysis shows distinct *SCGB1A1* and *SCGB1A1A* melting temperatures of 86.7°, and 87.6° respectively, indicating gene-specific amplification. M, 100-bp marker.

To gain insight into the role of *SCGB1A1* genes in the pathogenic mechanisms of RAO, we next sought to determine gene-specific expression in the lung, based on the previous observation that total *SCGB1A1* gene expression levels were reduced in affected animals [6]. Relative transcript levels were determined by quantitative RT-PCR (qRT-PCR) using gene-specific primers for *SCGB1A1* and *SCGB1A1A* (Figure 5A). Both equine GAPDH and 18S genes were amplified concurrently for use as internal standards, and all PCR products were analyzed by gel electrophoresis and melting curve analysis (Figure 5B-D). Lung tissues from clinically healthy horses and horses with RAO were assessed, and the latter group included individuals sampled during exacerbation and remission episodes. Results were reported as the ratio of *SCGB1A1A*/*SCGB1A1* gene expression.

**Figure 6:**
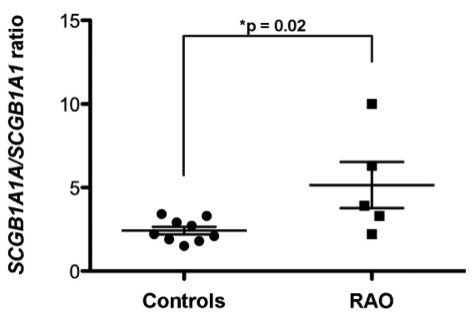
***SCGB1A1 *and *SCGB1A1A *expression analysis in lung tissues from control and RAO horses.** Quantification of *SCGB1A1* and *SCGB1A1A* RT-PCR products was relative to both equine *GAPDH* and ribosomal *18S* gene expression levels. Values are displayed as *SCGB1A1A*/*SCGB1A1* ratios. In control horses, the gene expression ratios were consistently and significantly lower compared to those of age-matched RAO horses (2.4 ± 0.2, n = 9 vs 5.1 ± 1.4, n = 5, respectively). Lung biopsies from RAO animals were from either exacerbation or remission periods.

As shown in Figure 6, similar expression ratios were detected in the lung of healthy individuals (2.4 ± 0.2, n = 9), who consistently had slightly higher *SCGB1A1A* than *SCGB1A1* expression. Comparable expression ratios were also noted in uterine tissues (2.6 ± 0.7, n = 7, data not shown), suggesting an equivalent distribution of the two *SCGB1A1* genes in different organs. However, the *SCGB1A1* expression ratio was significantly different (5.1 ± 1.4, n = 5) in RAO animals compared to control animals of similar age. Higher ratios were attributable to increased *SCGB1A1A* expression, suggesting that maintenance of an appropriate *SCGB1A1* gene ratio might be necessary for homeostasis, and that abrogation of the ratio may contribute to or reflect RAO development.

### SCGB 1A1 protein expression

**Figure 7:**
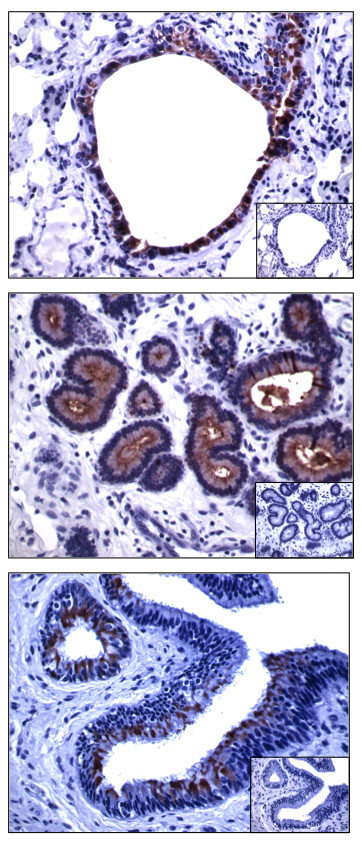
**Immunohistochemical identification of SCGB 1A1 proteins.** SCGB 1A1 proteins were detected in distal lung bronchioles (**A**), uterine glands (**B**), and Fallopian tube (**C**). Inserts are sections incubated with pre-immune serum instead of primary antibody. Note that not all cells within each epithelial layer express SCGB 1A1.

In order to evaluate the correlation between *SCGB1A1* gene expression and the distribution of the protein, different tissues were evaluated by immunohistochemistry using the previously described antibody to equine SCGB 1A1 [6]. This antibody was generated against a SCGB 1A1 peptide, which is shared by both SCGB 1A1 and SCGB 1A1A, and therefore was expected to label both predicted SCGB 1A1 proteins. Detailed examination of lung tissue revealed strong and specific SCGB 1A1 staining of the majority of non-ciliated cells lining the smaller bronchi and bronchioles (Figure 7A). The cytosolic signal was diffuse and did not show basal or apical predilection. SCGB 1A1 staining intensity was highest in the small bronchiolar ducts, suggesting greater expression in the distal bronchiolar tree. Diffuse faint staining was noted among intrabronchiolar secretions. Other pulmonary components such as blood vessels, fibroblasts, alveolar epithelium, goblet and basal cells, did not stain for SCGB 1A1.

Analysis of the extra-pulmonary tissues revealed that epithelial cells in the uterus, ovary, Fallopian tube and mammary gland also expressed SCGB 1A1. Within the uterus, large coiled glands composed of columnar epithelial cells stained most strongly positive (Figure 7B), while in the ovary only epithelium from the wall of residual cysts showed some degree of staining, and luteal cells were negative. The ciliated cells of the Fallopian tube stained positive, with a strong apical signal extending to the cilia (Figure 7C). Immunoreactive SCGB 1A1 was not detected in pancreas, liver, kidney, adrenal gland, large intestine, striated and smooth muscle, duodenum, adipose tissue, nerve, nose epithelium, skin, stomach, aorta, cartilage, bone marrow, brain and pituitary gland.

## Discussion

In this study we report the identification and characterization of three equine *SCGB1A1* genes. A large segment of genomic sequence was amplified for each *SCGB1A1* gene and the partial coding sequences leading to the mature secreted proteins were sequenced. We found that certain nucleotides from each of the three genes differed from the other two genes, and that this pattern was conserved amongst individuals. The distinct *SCGB1A1* genes were predicted to produce slightly different proteins, and were therefore referred to as *SCGB1A1P*, *SCGB1A1* and *SCGB1A1A* based on the recommended systematic gene nomenclature system [20]. A non-contiguous region composed of three distinct nucleotide variants was chosen as a signature sequence to generate assays specific for each individual *SCGB1A1* gene.

Analysis of the predicted *SCGB1A1* sequences extracted from EquCab2.0 (NCBI) revealed more than 90% pairwise identity between *SCGB1A1P*, *SCGB1A1*, and *SCGB1A1A* genes, including large segments of 5’- and 3’-flanking regions. This high level of sequence identity between both the coding and non-coding regions of *SCGB1A1* genes created a challenge for isolation and assessment of individual genes. Thus, more than 10 kb of non-coding sequence surrounding each *SCGB1A1* gene was interrogated to identify anchor regions for gene-specific primers. Primers selected included at least three nucleotides unique to each *SCGB1A1* gene. Sequence analysis of products confirmed specific amplification of desired targets with lack of cross-amplification.

Comparative analysis of the coding region of *SCGB1A1* genes revealed some disparity with the predicted EquCab2.0 sequences. Our sequences differed at three positions in *SCGB1A1P*, one position in *SCGB1A1*, and five positions in *SCGB1A1A*, corresponding to 1.1, 0.4, and 1.8% of difference, respectively. These differences may result from copy number variants and chromosomal rearrangements hindering automated sequence assembly of the equine genome [21]. This is consistent with recent studies reporting equine chromosome 12 as a “hotspot” for genomic rearrangements, such as enrichment in copy number variants and single nucleotide polymorphisms (SNP) [22,23]. A SNP was detected in *SCGB1A1P* and two SNPs in *SCGB1A1*, but neither affected the predicted translated products. Altogether, we identified several sequence differences to the EquCab2.0 genome, which highlighted the importance of developing gene-specific assays.

The three *SCGB1A1* genes had similar gene structure and highly conserved intron/exon organization, suggesting that each contained all the elements required for expression. Thus, the transcriptional state of each gene was evaluated by EPLD-PCR. *SCGB1A1* and *SCGB1A1A*, but not *SCGB1A1P*, were specifically detected in EPLD of lung and uterus. These results are consistent with detection of two differently migrating SCGB 1A1 proteins in uterine washes from mares in early pregnancy [18]. Furthermore, the sequence of each gene between start and stop codon was identical to our genomic sequence. The equine genome draft was derived from DNA of a single Thoroughbred mare, while our EPLD-PCR data were generated from animals of various genetic backgrounds and may thus be more representative of this challenging genome region.

*SCGB1A1P* transcripts were not detected in lung or uterine tissue. Reasons for lack of *SCGB1A1P* gene transcription were unclear, especially since the gene structure is virtually identical to that of the other *SCGB1A1* genes. However, the *SCGB1A1P* sequence was more variable between individuals, had a shorter promoter region (which may imply lack of regulatory elements) and contained a putative stop mutation in a significant percentage of individuals. These characteristics suggest that *SCGB1A1P* may be a pseudogene.

The non-synonymous nucleotide variations observed between *SCGB1A1* and *SCGB1A1A* result in 12 amino acid (AA) substitutions among the 70 residues of the mature secreted proteins. Seven of the variable AAs are concentrated between position 26 and 36 (protein including the signal peptide). This region borders the SCGB 1A1 central cavity that binds hydrophobic ligands (reviewed in [24]), and AA with hydrophobic properties comprise the cavity [25]. Since some conserved AA, such as phenylalanine 27 (F27), also have ligand-binding properties, substitution of F27 to L27 in SCGB 1A1A suggests 1) a change in ligand-binding specificity, and 2) that SCGB 1A1 and 1A1A may have independently evolved to bind distinct substrates. Other substitutions in this region largely maintain hydrophobic properties (A28 to V28, I31 to V31, G33 to A33, F35 to Y35), implying minor change in ligand affinity. There is high sequence identity in other regions of each protein with preservation of critical structural residues such as C24 and C90 needed for homodimer interaction, and K63, D67 and A58 required for protein stability. However, also of interest, the predicted isoelectric point (pI) of SCGB 1A1 and 1A1A proteins are 5.1 and 6.3, respectively, which corresponds to the SCGB1A1 variants described in uterine washes of pregnant horses [18].

The spectrum of tissues expressing *SCGB1A1* has not been extensively studied in horses. Expression nonspecific for individual genes was previously identified in lung, uterus and prostate, and absent in liver, kidney, heart, spleen, as well as thyroid, pituitary and adrenal gland tissues by Northern blot analysis [11]. We selected a gene-specific RT-PCR approach to characterize the distribution of *SCGB1A1* and *SCGB1A1A* transcripts in a total of 33 tissues. This assay amplified the coding region of the entire mature protein (second and third exons) to reduce potential genomic DNA targeting, and specificity was verified by random purification and sequencing of amplicons. As expected, *SCGB1A1* and *SCGB1A1A* transcripts were strongly detected in lung and uterus, but also in Fallopian tube and mammary gland tissue. Fewer transcripts of either gene were present in brain, pituitary gland, eye, nose epithelium, tongue, salivary gland, trachea, aorta, liver, spleen, small and large intestines, adrenal gland, kidney, skin, bladder, urethra, prostate, epididymis, seminal vesicle, testis and ovary, and no transcripts were detected in eyelid gland, thyroid, bone marrow, cardiac muscle, pancreas, stomach and lymph node. Detection of transcripts in a greater range of tissues by PCR than by Northern blotting may reflect higher sensitivity of the former, and greater specificity due to exact primer match. Some tissues had more of one relative to the other gene product, suggesting tissue-specific expression patterns. While the regulatory mechanisms that could selectively drive the expression of individual *SCGB1A1* genes remain to be elucidated, this finding is consistent with unique functions of different SCGB 1A1 proteins.

Immunohistochemical analyses were carried out to evaluate the correlation of *SCGB1A1* gene and protein expression, and to determine cell-specific expression within tissues. The antibody employed recognized an epitope shared by all SCGB 1A1 proteins. Strong staining was detected in tissues expressing the highest number of transcripts such as lung, uterus, Fallopian tube and mammary gland. However, within these tissues, SCGB 1A1 was present in only specific epithelial cell populations, and absent in all other cell types (Figure 7). This distribution likely contributed to variation of *SCGB1A1* transcript intensity, since the proportion of epithelial cells and subtypes in tissues selected was variable [26].

SCGB 1A1 may be selectively taken up and targeted for degradation upon binding to a transmembrane protein called cubilin [27]. Interaction of SCGB 1A1 with lipocalin-1 interacting membrane receptor (LIMR) has also been reported, but the effect of this interaction is unclear [28]. Of note, LIMR is expressed in tissues positive for *SCGB1A1* transcripts, including lung, mammary gland, trachea, prostate, testis, pituitary gland, adrenal gland, cerebellum, kidney, and colon [29]. SCGB 1A1 protein was not detected in tissues with low transcript expression such as bladder, trachea, liver, nose epithelium, pituitary and intestine. Possible reasons are lesser sensitivity of IHC compared to PCR, and transient gene expression insufficient to produce detectable protein, as has been reported in studies of rabbit tissues [30].

Horse and human have a similar pattern of *SCGB1A1* transcript distribution in lung [31], uterus [13,32], Fallopian tube [33], prostate [34,35], trachea, thyroid, mammary gland, brain, pituitary, thymus, aorta, heart, stomach, spleen, adrenal gland, kidney, liver, small intestine, ovary and testis [36]. In humans, the *SCGB1A1* gene was ultimately considered to be ubiquitously expressed in most cells of epithelial origin and to inactivate inflammatory mediators on surfaces exposed either directly or indirectly to the external environment [37,38]. Conversely, it was also reported that decreased *SCGB1A1* expression could contribute a tumor microenvironment permissive of inflammation and hence tumor progression [38,39].

Since total *SCGB1A1* expression was reported as decreased in RAO, we sought to evaluate if both *SCGB1A1* and *SCGB1A1A* genes were similarly affected. Our analysis demonstrated that the ratio of *SCGB1A1A*/*SCGB1A1* was significantly different in RAO affected-animals compared to controls. This finding may arise as a result of chronic inflammation with preferential transcription of *SCGB1A1A*, or may signal inherent differences in transcriptional regulation of *SCGB1A1* genes. Segregation analysis previously revealed a complex genetic background influencing expression of the RAO phenotype [40]. *SCGB1A1* was among candidate genes [10], suggesting that further evaluation of specific gene expression may be warranted. The latter observation also raises the question whether *SCGB1A1* genes are controlled by different regulatory mechanisms and whether they have different physiological functions. Such hypotheses are difficult to address due to the absence of multiple *SCGB1A1* copies in other mammals except other equidae, such as donkeys and Przewalski horses (unpublished data). *SCGB1A1* isoforms in equid species remain to be characterized, but may yield insight into *SCGB1A1* gene origin and ancestral gene triplication.

## Conclusions

Three equine *SCGB1A1* genes were isolated and characterized. *SCGB1A1P* appears to have evolved into a pseudogene, which no longer generates a detectable transcript and includes a non-sense mutation in the majority of animals. The distribution of *SCGB1A1* and *SCGB1A1A* gene transcripts and proteins indicates highly specific expression in specialized epithelial cells of lung and reproductive organs. Gene specific assessment of transcripts showed approximately 2.5 fold higher expression of *SCGB1A1A* than *SCGB1A1* in lung and uterus of control animals, and an increased ratio in lung tissue of animals with RAO, an asthma-like condition. Future studies will assess the function of different SCGB 1A1 proteins and attempt to elucidate their anti-inflammatory properties.

## Methods

### Samples

Animal procedures were approved by the University of Guelph Animal Care Committee (Protocol R10-031) and conducted in compliance with guidelines of the Canadian Council on Animal Care. All horses belonged to the institutional research herd; horses with and without RAO were of similar age ranging from 12 to 20 years. Horses with RAO had a history of recurrent cough, difficulty with exhaling air, and neutrophilic inflammation in BAL fluid samples. Control horses had no history of lung disease, had normal physical exam findings, and no abnormalities on airway bronchoscopy or pulmonary function testing, as described before [6].

Horses were restrained in stocks and percutaneous lung biopsies were obtained under sedation with romifidine (10 mg/mL, IV), as previously described [6]. Samples were immersed in RNAlater™ solution (Qiagen, Mississauga, ON) and stored at -80°C until RNA preparation.

### *SCGB1A1* gene identification and localization

*SCGB1A1* sequences were obtained from the National Center for Biotechnology Information (NCBI) database. Alignment of the *Equus caballus* chromosome 12 genomic contig (EquCab2.0) [GenBank: NW_001867370.1] [19] and the equine secretoglobin precursor mRNA [GenBank: AY885564.1] was performed through a nucleotide megablast analysis for highly similar sequences using the following parameters: expected threshold, 10; word size, 16; match-mismatch scores, 1, -2; gap cost - linear; filter - low complexity. Further data processing, including sequence extractions, multiple sequence alignments, and statistical analysis, were accomplished with Geneious Pro software (version 5.5.3, Auckland, NZ) with occasional minor manual adjustments [41].

### Isolation and characterization of *SCGB1A1* genomic sequences

Blood samples or buccal swabs were available from 24 adult horses including 6 breeds (Standardbred, Thoroughbred, Icelandic, Canadian sport, Lipizzaner, and Quarter horses) and animals of mixed breeding. Genomic DNA was extracted according to the manufacturer’s protocol (DNA Mini kit, Qiagen). LR-PCR primers were as follows: *SCGB1A1P* primer forward UG1-F (5′-ACA GAG CCA GCC CAA GCA ATG-3′) and reverse UG1-R (5′-GAT TAC CTT GGC GGT TGC CTA GAG-3′), *SCGB1A1* primer forward UG2-F (5′-CAC CTA ACA GCC TCA TCT C-3′) and reverse UG2-R (5′-GTG AGA GCT CTC ATC TGG TA-3′), and *SCGB1A1A* primer forward UG3-F (5′-ACA CAG ATC TGA TGC CCA AG-3′) and reverse UG3-R (5′-AGT GCA GCT CTC TCA GGC AT-3′), amplifying 5559, 6029, and 5442 bp of genomic DNA, respectively. Primers were obtained from Sigma-Aldrich (Burlington, ON). LR-PCR amplifications were carried out using a Platinum Taq polymerase PCR kit (Invitrogen, Mississauga, ON). Each reaction was performed in a final volume of 50 μL, including 5 μL of 10X PCR buffer, 0.2 mM dNTPs, 2 mM MgSO_4_, 0.3 μM of each primer, 2 U of Platinum Taq, and 5 μL of template DNA (100 ng). Conditions for amplification were 1 min at 94°C followed by 35 cycles of 94°C for 30 s; gene-specific annealing T°C for 30s; and 68°C for 6:30 min, followed by final elongation for 10 min at 68°C. Gene-specific annealing temperatures for *SCGB1A1-1*, *-2*, and *-3* were 62°C, 55°C and 60°C respectively. Twenty μL of each PCR product was separated by electrophoresis in a 1% agarose gel stained with SYBR Safe (Invitrogen). The amplified DNA bands were cut out and the DNA was extracted, purified (QIAquick, Qiagen) and quantified using a NanoDrop 2000 photometer (Thermo Fisher Scientific, Mississauga, ON). To validate the gene-specificity of the PCR products, each purified DNA fragment was digested with *HindIII* (Invitrogen), separated by electrophoresis and monitored for the appropriate digestion pattern.

A genomic DNA band of 526 bp coding for the full-length mature secreted protein (exon 2 to 3) was amplified by nested PCR using LR-PCR purified DNA products as template and the forward UGn-F (5′-GCT TCT GCA GRA ATC TGC CAG AG-3′) and reverse UGn-R (5′-CTA AGC ACA CAG TGG GCT CTY TRC-3′) primers. PCR amplifications were carried out in duplicate using the Taq DNA polymerase Native PCR kit (Invitrogen) in a final volume of 25 μL of PCR buffer (2 μL of 10X PCR buffer, 1.0 mM dNTP, 1.5 mM MgSO_4_, 0.6 μM forward and reverse primers, 1 unit Platinum Taq, and 1 μL of template DNA). Cycling conditions were 1 min at 94°C followed by 30 cycles of 94°C for 30 s; 62°C for 30 s; and 72°C for 90 s with a final elongation of 7 min at 72°C. Twenty μL of each PCR product was subjected to electrophoresis; bands of appropriate size were excised from the gel, purified and submitted for automated sequencing (Laboratory Services Division, Guelph, ON). Amplicons were analyzed in duplicate using reverse and forward primer sequencing strategies. The consensus sequence for each copy was determined using multiple sequence alignments.

### Detection of distinct *SCGB1A1* transcripts

Total RNA was isolated from fresh or frozen lung and uterus tissues (RNeasy, Qiagen) according to the manufacturer′s recommendations. RNA integrity was verified through capillary electrophoresis in a 2100 Bioanalyzer (Agilent Technologies) prior to analysis. Complementary DNA (cDNA) was synthesized following the Superscript III Reverse Transcriptase kit′s protocol (Invitrogen), with an additional 15 min DNAse I treatment (Qiagen) at room temperature. A cDNA band of 256 bp delimited by the start (ATG) and termination (TAG) codon within each of the *SCGB1A1* genes was targeted with the following primers: forward UGm-F (5′-GTC CAC CAT GAA ACT CGC CA-3′) and UGm-R (5′-CTA AGC ACA CAG TGG GCT C-3′). End-point limiting dilution (EPLD)-PCR assays were performed in a volume of 25 μL of PCR reaction mix (2 μL of 10X PCR buffer, 1.0 mM dNTP, 1.5 mM MgCl_2_, 0.6 μM forward and reverse primer, 1 unit of Platinum Taq and 1 μL of template cDNA). A broad range of serially diluted cDNA concentrations was tested (0.25 to 0.000156 ng/μL) to determine the optimal limiting-dilution for each sample (0.0005 to 0.0006 ng/μL). PCR products were separated by electrophoresis, purified, sequenced and identified via their *SCGB1A1* gene-specific signature sequences.

### Tissue-specific expression of *SCGB1A1*

*SCGB1A1* and *SCGB1A1A* relative transcript levels were evaluated by semi-quantitative reverse transcriptase-PCR (RT-PCR) in 33 different tissues from normal adult horses including cortical brain, pituitary, eye, nose epithelium, tongue, thyroid, trachea, lung, aorta, cardiac muscle, liver, spleen, small and large intestine, stomach, kidney, pancreas, skin, bladder, urethra, prostate, epididymis, seminal vesicle, testis, uterus, ovary, Fallopian tube, bone marrow, lymph node, as well as mammary, salivary, adrenal and eyelid glands. Total RNA isolation and cDNA synthesis were performed as described above. A specific cDNA band of 200 bp was amplified for each *SCGB1A1* gene using the following primers: *SCGB1A1* forward UGrt-2 F (5′-GCT TTG CAG ACA TCA TTC AAG GCC-3′) and reverse UGrt-2R (5′-CTA AGC ACA CAG TGG GCT CTT TG-3′) as well as *SCGB1A1A* forward UGrt-3 F (5′-GAT TKG TAG GCA TCG TTC AAG CCC-3′) and reverse UGrt-3R (5′-CTA AGC ACA CAG TGG GCT CTC TA-3′). A 254 bp equine glyceraldehyde dehydrogenase (*GAPDH*) gene product served as an internal control using forward GAP-F (5′-GTT TGT GAT GGG CGT GAA CC-3′) and reverse GAP-R (5′-TTG GCA GCA CCA GTA GAA GC-3′) primers. PCR amplifications were carried out using the HotStar Taq Plus PCR kit (Qiagen) in a 20 μL Master mix (10 μL of 2X PCR buffer, 0.4 μM of copy-specific forward and reverse primers, and 1 μL of template cDNA). Conditions for amplification were 5 min at 95°C followed by 30 cycles of 95°C for 30 s; 61°C for 30 s; 72°C for 1 min followed by a final elongation of 7 min at 72°C. PCR products were analyzed by electrophoresis in a 1% agarose gel and stained with SYBR Safe. PCR-grade water was distributed as template in negative control reactions. No amplifications were detected in samples with water instead of template DNA.

### Quantification of *SCGB1A1* and *SCGB1A1A* gene expression

*SCGB1A1*, *SCGB1A1A* and *GAPDH* primers were as described above. The equine *18S* ribosomal RNA gene was retained as an additional internal control using forward 18S-F (5′- ATG CGG CGG GGT TAT TCC-3′) and reverse 18S-R (5′-GCT ATC AAT CTG TCA ATC CTG TCC-3’) primers. Quantitative PCR amplifications were performed in a Master mix containing 10 μL of SYBR Green 2X PCR buffer (Qiagen), 8 μL of PCR-grade water, 0.4 μM of each forward and reverse primer, and 1 μL of cDNA template (1 ng/μL). Conditions for amplification were 7 min at 95°C followed by 45 cycles of 95°C for 15 s; 61°C for 15 s; 72°C for 20 s using a LightCycler**®** 480 instrument (Roche, Montreal, QC). *SCGB1A1* primer specificity and identity of the PCR products was confirmed with a melting curve (95°C for 5 s; 45 to 95°C; 40°C for 10 s) and sequence analysis, respectively. For each gene, a series of purified cDNA PCR product dilutions (100, 10, 1, 0.1, 0.01, 0.001 ng/μL) was amplified and the average crossing point of each dilution was used to derive a standard curve. *SCGB1A1*, *SCGB1A1A*, *GAPDH*, and *18S* cDNA were amplified in triplicate for each sample along with standard curve calibrators. Data were analyzed using LightCycler**®** 480 SW 1.5 software (Roche). Statistical analysis was carried out using Prism5 (GraphPad Software, San Diego, CA).

### Immunohistochemistry

Expression of SCGB 1A1 protein was investigated through immunohistochemical staining of the 33 adult horse tissues described above. Fresh tissues were fixed in 10% neutral buffered formalin overnight, embedded in paraffin, and sectioned to 5-μm thickness. Sections were deparaffinized in xylene, rehydrated in graded alcohols and incubated consecutively for 10 min in Dako endogenous dual enzyme blocker (DakoCytomation), 30 min with Dako protein block (serum-free), 30 min with SCGB 1A1 primary antibody (1:400 dilution), and 30 min with horseradish peroxidase-labeled secondary IgG (1:2000, Dako EnVision HRP). Bound antibodies were detected with Nova Red (Vector Laboratories, Burlington, ON) chromogen, and slides were counterstained with hematoxylin. SCGB 1A1 primary antibody was raised in a rabbit immunized with a 21mer peptide [6]. Pre-immune rabbit serum was used for negative control slides. Images were acquired on a Leica DMRA2 microscope (Leica Microsystems, Concord, ON) using Open Lab software (PerkinElmer, Waltham, MA).

### Statistical analysis

Values were expressed as means ± standard deviations. Unpaired two-sample Student’s *t*-test was used for statistical analysis; *p* ≤ 0.05 was considered significant.

## Abbreviations

EPLD-PCR: End-point limiting dilution PCR; LR-PCR: Long-range PCR; RAO: Recurrent airway obstruction; SCGB: Secretoglobin; sqPCR: Semi-quantitative PCR.

## Competing interests

The authors declare no competing financial or non-financial interests.

## Authors’ contributions

OC generated the PCR assays and sequences, analyzed the data and wrote the manuscript. BL and AH procured tissues, and interpreted morphological findings and immunohistochemical staining. MC optimized and performed immunohistochemical staining; LB assisted with tissue specific semi-quantitative expression analysis; PK and LV characterized horses with RAO, obtained in vivo samples, and designed peptides for antibody generation; DB conceived the study, assisted with data analysis, microscopic review, and image preparation; and revised the manuscript drafts. All authors read and approved the final manuscript.

## Supplementary Material

Additional file 1 **Figure S1.** Multiple sequence alignment of *SCGB1A1P*, *SCGB1A1* and *SCGB1A1A* cDNA consensus sequences. The sequences displayed are as follows: predicted *SCGB1A1P* EquCab2.0, the *SCGB1A1P* variant A (JQ951929), variant B (JQ951930) and variant C (JQ951931) derived in this study (genomic DNA); predicted *SCGB1A1* EquCab2.0, *SCGB1A1* variant A (JQ906259) and variant B (JQ906260) determined in this study (cDNA); predicted *SCGB1A1A* EquCab2.0 and *SCGB1A1A* (JQ906261) derived in this study (cDNA). For each cDNA sequence, the corresponding protein sequence is displayed beneath. Colored annotations highlight discordant bases and amino acids relative to the consensus sequence (colored) at the top of the figure. Click here for file
