# Supplementary figures and images for: Multiple secretoglobin 1A1 genes are differentially expressed in horses
Source: BMC Genomics. 2012 Dec 19;13:712. doi: 10.1186/1471-2164-13-712 (PMC3556144; doi:10.1186/1471-2164-13-712)

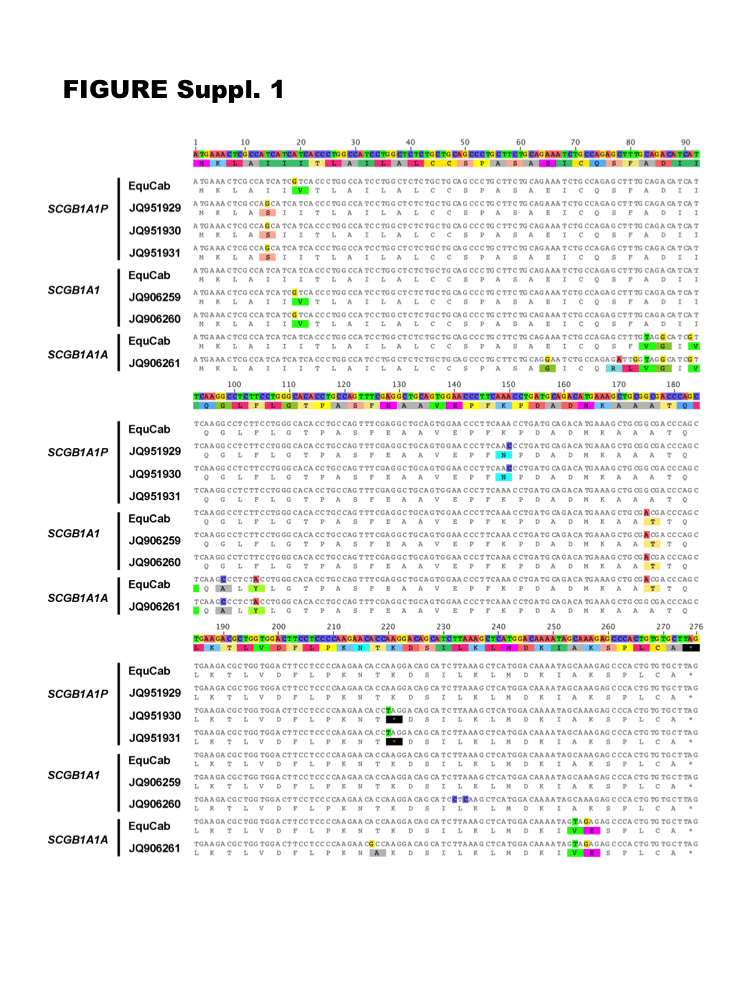

Supplement: Additional file 1 — Figure S1. Multiple sequence alignment of SCGB1A1P, SCGB1A1 and SCGB1A1A cDNA consensus sequences. The sequences displayed are as follows: predicted SCGB1A1P EquCab2.0, the SCGB1A1P variant A (JQ951929), variant B (JQ951930) and variant C (JQ951931) derived in this study (genomic DNA); predicted SCGB1A1 EquCab2.0, SCGB1A1 variant A (JQ906259) and variant B (JQ906260) determined in this study (cDNA); predicted SCGB1A1A EquCab2.0 and SCGB1A1A (JQ906261) derived in this study (cDNA). For each cDNA sequence, the corresponding protein sequence is displayed beneath. Colored annotations highlight discordant bases and amino acids relative to the consensus sequence (colored) at the top of the figure. [file 1471-2164-13-712-S1.tiff]
